# Supplementary material for: Breaking the Boundaries of the Goldschmidt Tolerance Factor with Ethylammonium Lead Iodide Perovskite Nanocrystals
Source: ACS Nano. 2024 Dec 26;19(1):1557–65. doi: 10.1021/acsnano.4c14536 (PMC11752489; doi:10.1021/acsnano.4c14536)
Supplement: Supplementary file 1 — nn4c14536_si_001.pdf [file nn4c14536_si_001.pdf]

Supporting Information for

Breaking the Boundaries of the Goldschmidt Tolerance Factor with Ethylammonium Lead Iodide Perovskite Nanocrystals

C. Meric Guvenc[1,2], Stefano Toso[2], Yurii P. Ivanov[3], Gabriele Saleh[2], Sinan Balci[4], Giorgio Divitini[3], Liberato Manna[1]*

[1] Department of Materials Science and Engineering, İzmir Institute of Technology, 35433 Urla, İzmir, Turkey

[2] Nanochemistry, Istituto Italiano di Tecnologia, Via Morego 30, Genova, Italy

[3] Electron Spectroscopy and Nanoscopy, Istituto Italiano di Tecnologia, Via Morego 30, Genova, Italy

[4] Department of Photonics, İzmir Institute of Technology, 35433 Urla, İzmir, Turkey

Contents

S1. Supplemental figures and tables……………………………………………………….S2

S2. $EAPbI_3/PbI_2$ epitaxial interface modeling……………………………………………..S9

S3. $EAPbI_3$ nanoplatelets analysis……………………………………………………..S15

S4. References………………………………………………………………………………S18

S1. Supplemental figures and tables

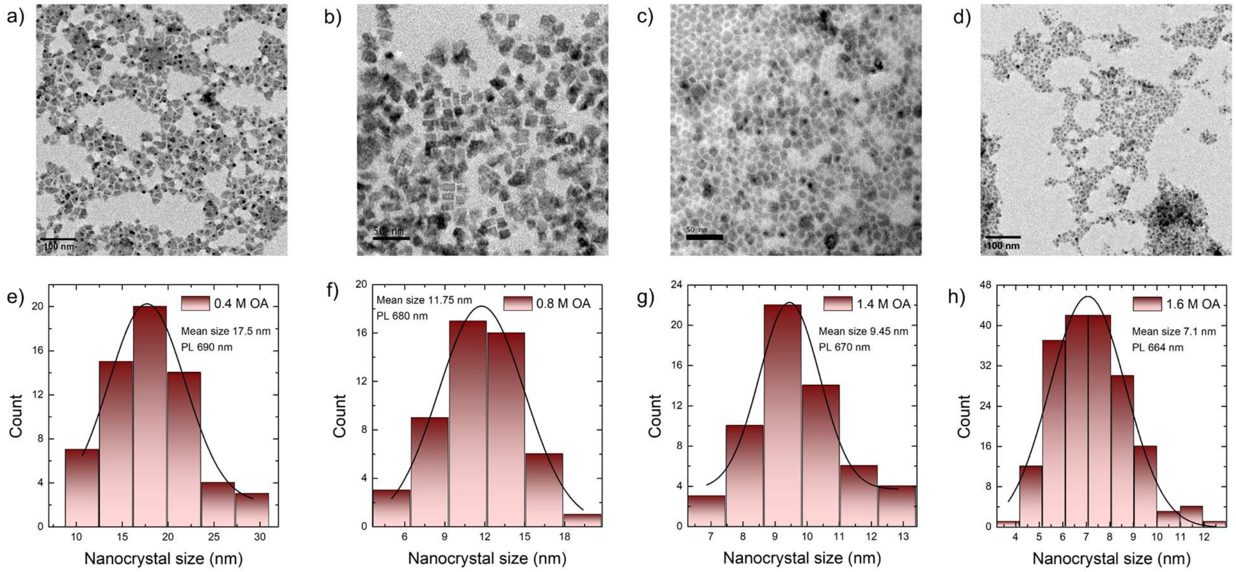

Figure S1. (a, b, c, d) TEM images of the $EAPbI_3$ perovskite nanocrystals, which contain 0.4 M, 0.8 M, 1.4 M, and 1.6 M OA in the final solution, and (d, e, f, h) their related particle size distributions, respectively.

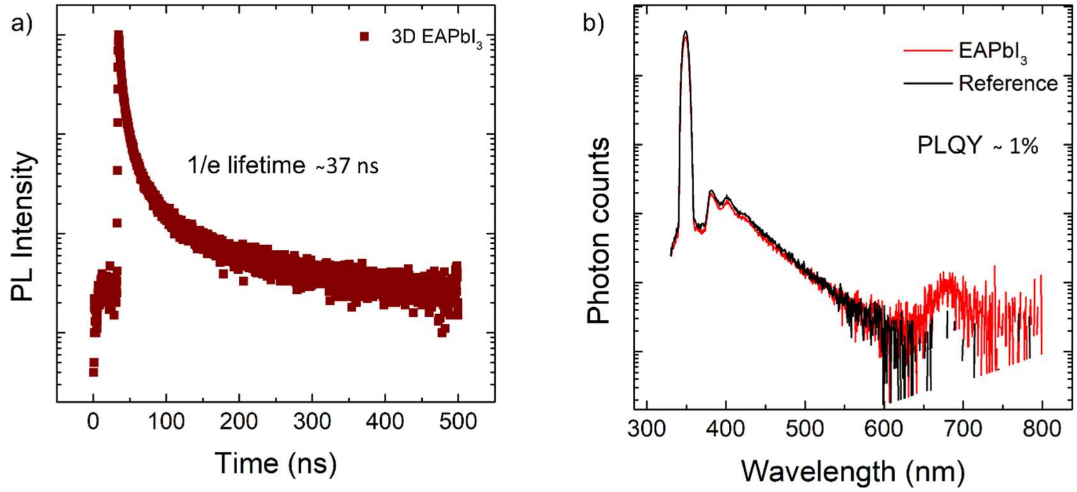

Figure S2. (a) PL intensity decay curve and (b) spectra recorded for PLQY measurement of $EAPbI_3$ perovskite nanocrystals.

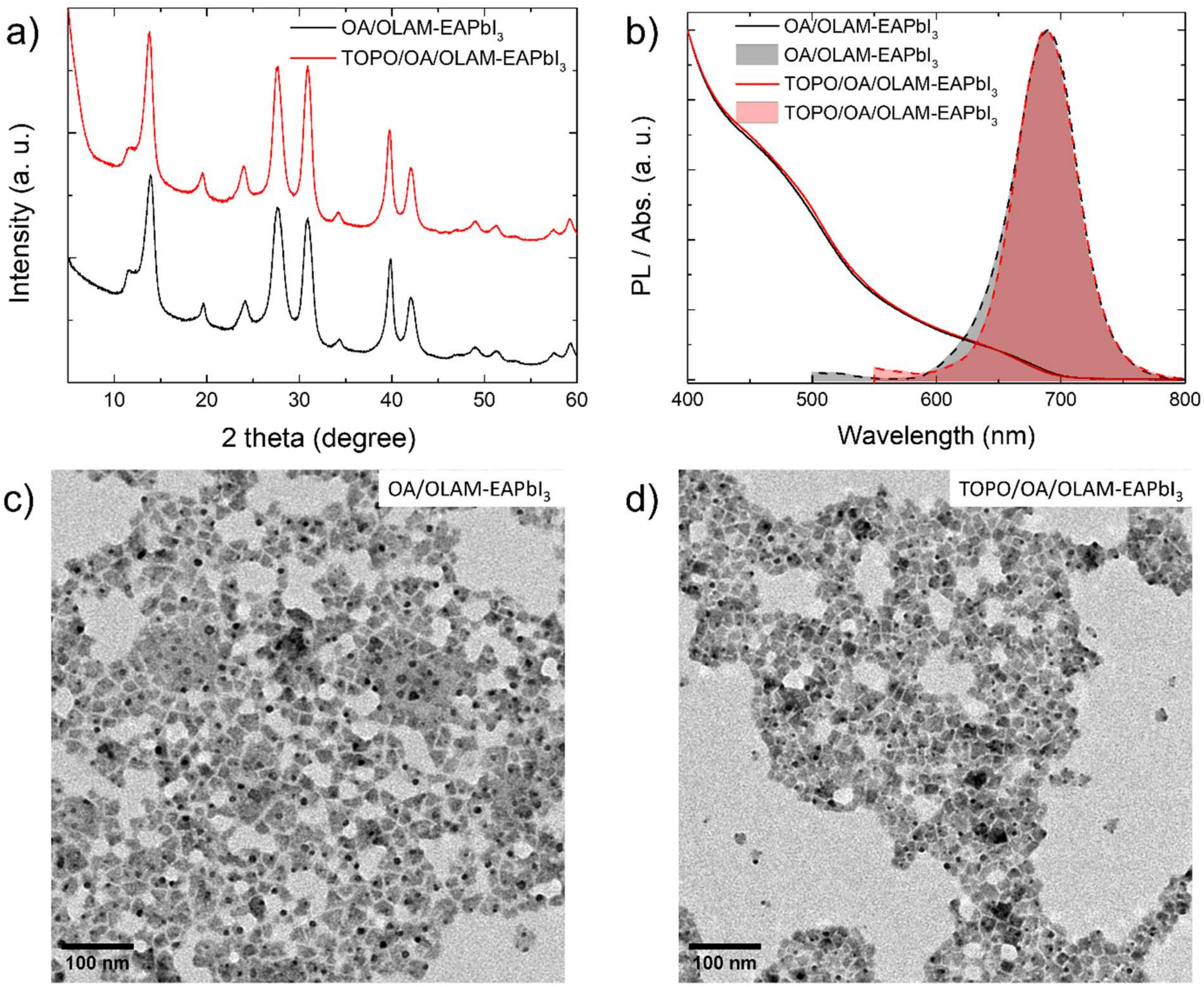

Figure S3 Comparison of the absorption, PL spectra, TEM images, and XRD patterns of the $EAPbI_3$ NCs synthesized with two methods used in this study. XRD patterns, absorption, PL spectra, and morphology of the $EAPbI_3$ NCs are very similar for both methods.

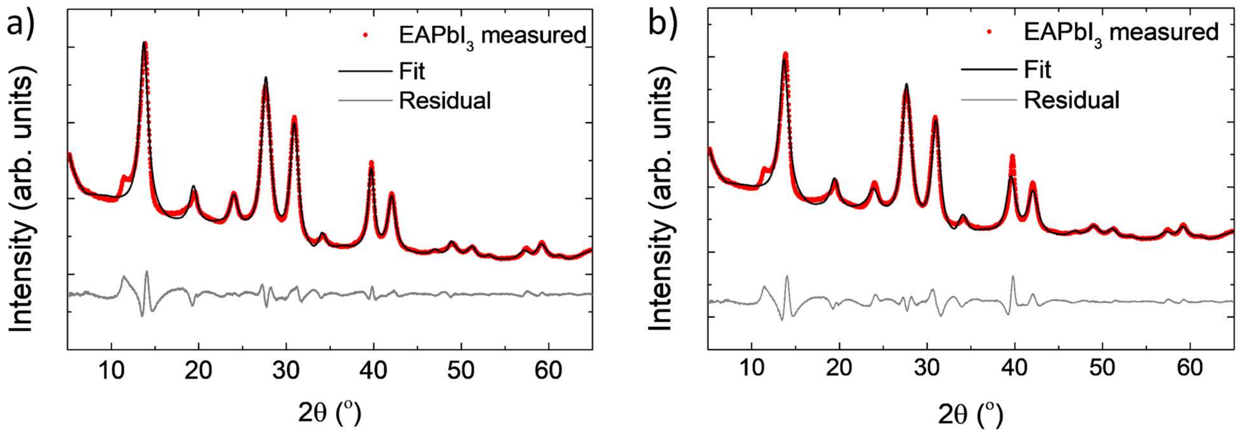

Figure S4. Experimental XRD data of the $EAPbI_3$ perovskite fitted with (a) R-3c and (b) Pm-3m prototype.

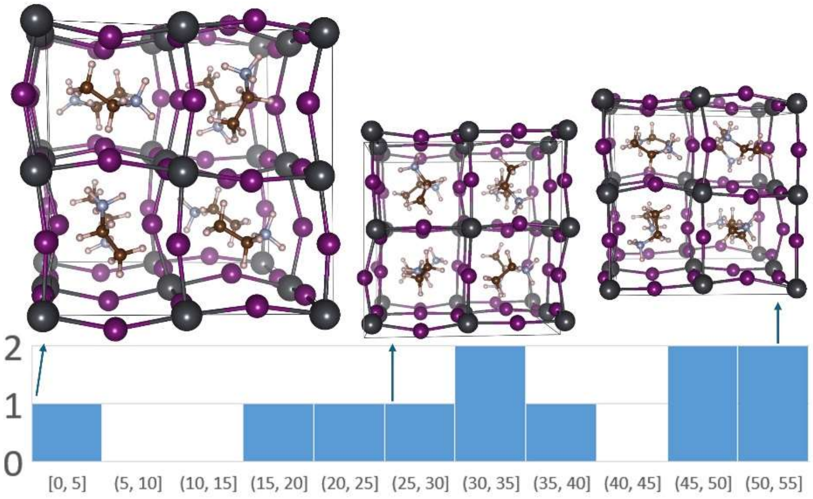

Figure S5 Histogram showing the energy distribution (meV/formula unit) of the 11 $EAPbI_3$ configurations obtained through simulated annealing. Representative configurations are shown. the larger, left most one is the lowest-energy structure (putative structural ground state) obtained. Color code for atoms as follows. Pb= grey, I=violet, C=brown, N=blue, H=white.

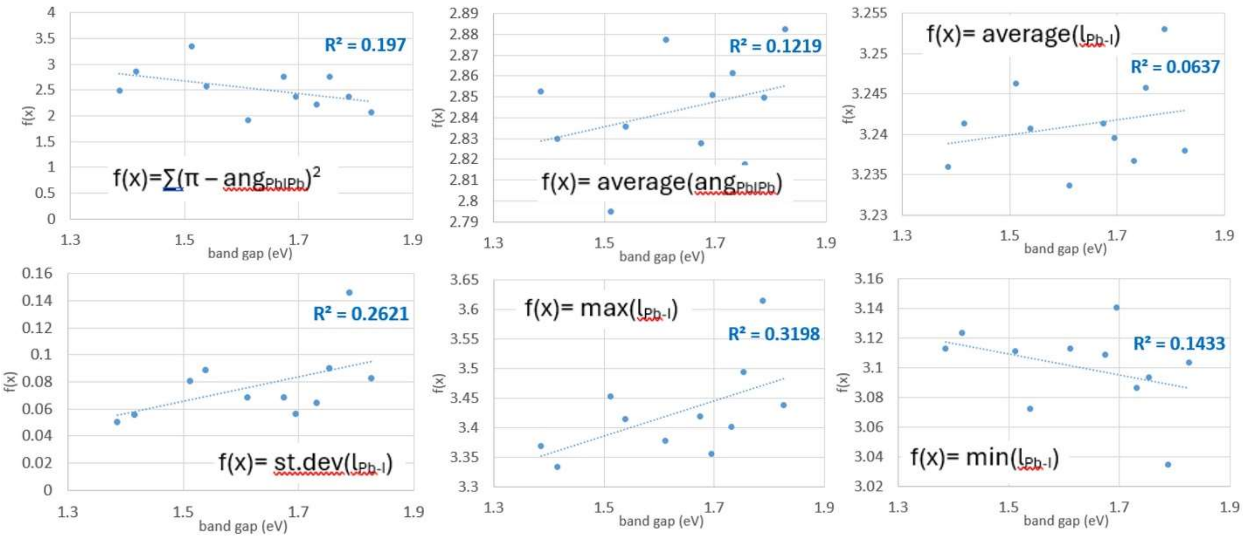

Figure S6 Plots of the band gap vs various measures of Pb-I sublattice distortion for the 11 studied $EAPbI_3$ configurations. For each plot, the function reported on the vertical axis is shown in black inside the plot. ang_{PbIPb} is the angle formed by Pb with two bonded I atoms, and $l_{Pb\text{-}I}$ is the Pb-I bond length. The Pearson correlation coefficient squared (R^2) is reported in blue in each plot.

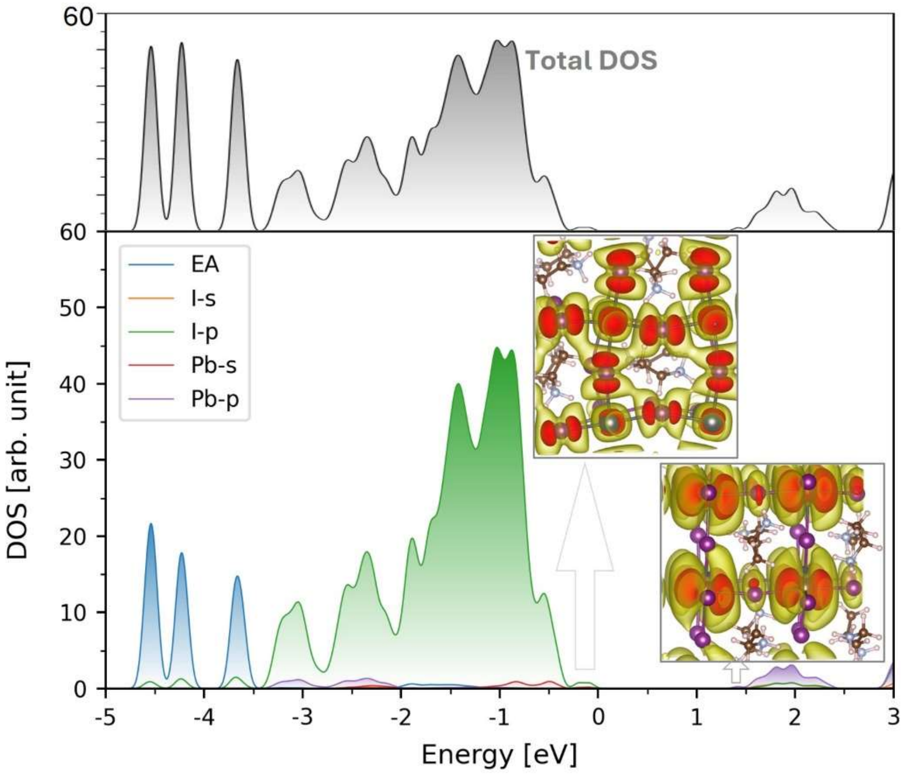

Figure S7 Density of states (DOS) and partial charge density for $EAPbI_3$ (lowest-energy configuration). The top plot reports the total DOS, while the bottom plot shows the atomic orbital-projected contributions, color legend on the top left. The charge density corresponding to the valence band maximum (from -0.25 eV to Fermi level) and conduction band minimum (from Fermi level to + 1.5 eV) are reported in the insets as yellow and red isosurfaces (isovalues, in VASP units: $5*10^{-5}$ and $5*10^{-4}$ for valence and $1*10^{-5}$ and $1*10^{-4}$ for conduction). Color code same as in Fig. S5.

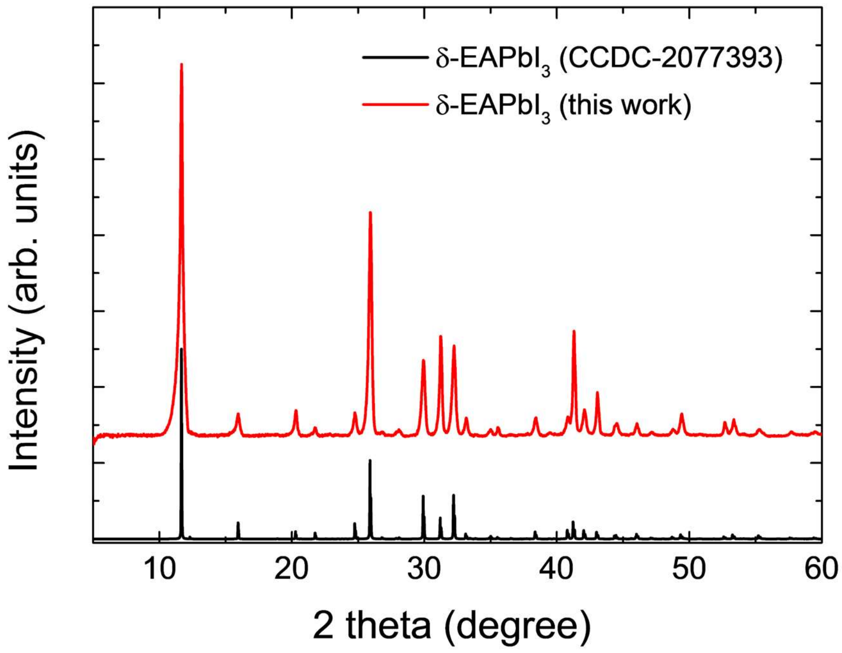

Figure S8. Comparison of the non-perovskite δ-$EAPbI_3$ crystals synthesized in this work and reference δ-$EAPbI_3$ (CCDC-2077393) XRD patterns.[1]

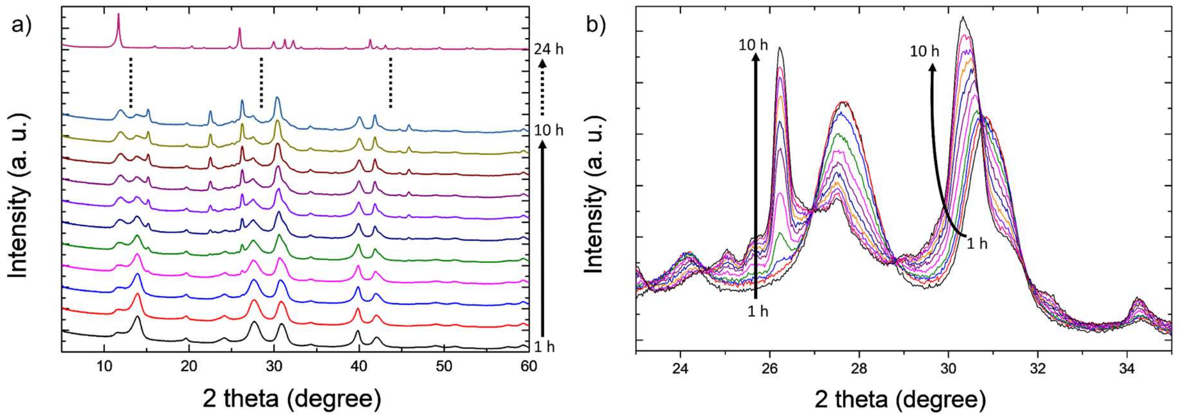

Figure S9. (a, b) The time-dependent XRD measurements of the $EAPbI_3$ perovskite nanocrystals. XRD analysis demonstrates that phase transformation starts after 2 hours in the drop-cast films of the $EAPbI_3$ nanocrystals under ambient conditions. After 24 hours, the $EAPbI_3$ perovskite phase completely transforms to δ-$EAPbI_3$ phase. (b) It is obvious that peak intensities change even 10 hours later, and phase transformation was not complete.

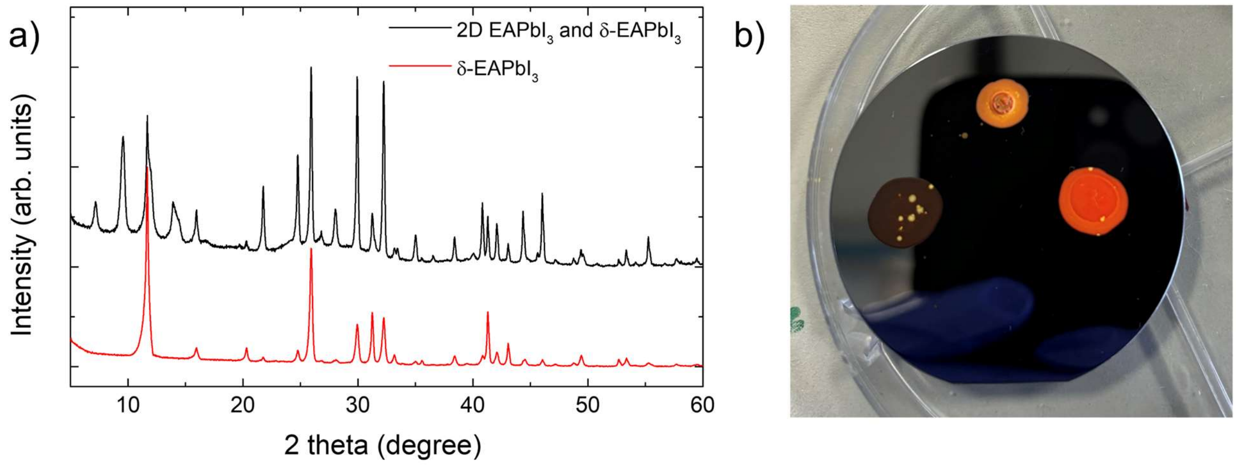

Figure S10. (a) Transformation of the $EAPbI_3$ perovskite phase to both 2D structures and the non-perovskite δ-EAPbI3 simultaneously. Repetitive peaks at the low angle indicate the formation of 2D structures. Further, peaks at 11° and 26° indicate the formation of δ-$EAPbI_3$ in the black line. The red line demonstrates pure δ-$EAPbI_3$. (b) Digital images of the drop cast films in the case of the transformation from $EAPbI_3$ perovskite phase to 2D and δ-$EAPbI_3$.The film's color was changed from black to orange. Also, it is possible to see some yellow spots related to δ-$EAPbI_3$ formation in the digital image.

S2. $EAPbI_3/PbI_2$ epitaxial interface modeling

The epitaxial relations between $EAPbI_3$ and PbI_2 were identified using the Ogre Python library for the prediction of epitaxial interfaces.[2–4] For simplicity, we adopted a pseudocubic description for $EAPbI_3$, while for PbI_2 we selected the trigonal reference structure proposed in previous study.[5] Since the relative orientation of the two domains could be inferred from the HAADF-STEM images, we did not perform a full exploration of the possible epitaxial relations. Instead, we used Ogre to identify the 2D-supercells describing the two observed epitaxial interfaces (Figure S11) and to propose an optimized structure model thereof.

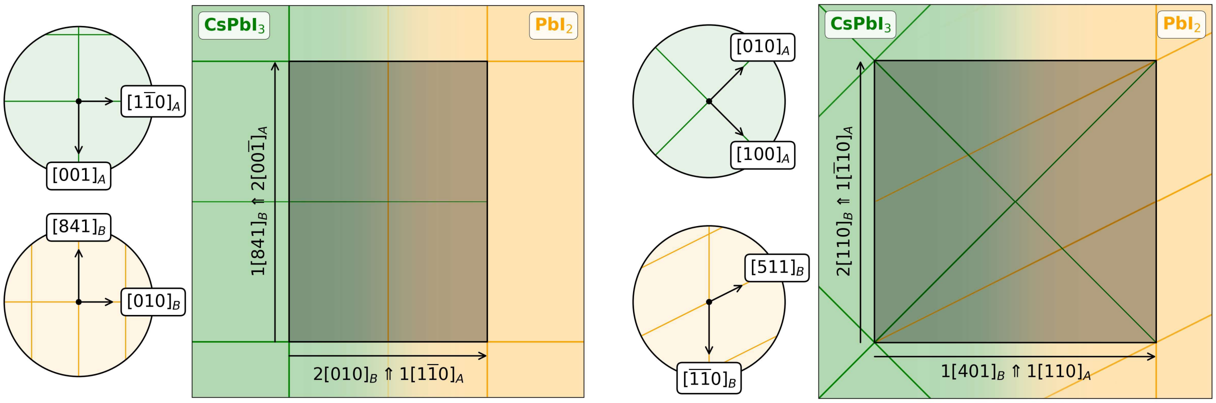

Figure S11. Interface supercells and relative orientation of the $EAPbI_3$ and PbI_2 lattices (in green and orange respectively) for the epitaxial interfaces shown in Figure 2e (left) and Figure 2f (right). The first interface occurs between the (110) plane of $EAPbI_3$ and the (10-8) plane of PbI_2, and is characterized by a supercell area of 116 Å^2 and 6 % strain. The second interface occurs between the (001) plane of $EAPbI_3$ and the (-114) plane of PbI_2, and is characterized by a supercell area of 82 Å^2 and 6 % strain. We note that this second epitaxial relation is equivalent to the one reported in Ref.[5] or the $PbI_2/FAPbI_3$ system. See Ref.[2] for details on the interpretation of these diagrams.

To perform the interface model optimization, we replaced EA^+ in the structure with Cs^+, because the version of Ogre we employed does not support structures involving organic cations. However, the lattice constant was kept to the 6.4 Å value we measured for $EAPbI_3$, and because the Ogre code adopts a fully classical electrostatic potential for energy evaluation, replacing one monovalent cation for another is expected to have limited impact on the outcome of simulations.

For both interfaces the structure proposed by Ogre was describing adequately the position of heavy atoms visible in the HAADF-STEM images reported in Figure 2. Notably, both models appear well connected, with the I^- anions of $EAPbI_3$ bonding naturally with the Pb^{2+} cations of PbI_2 so to complete their octahedral coordination environment.[2] Note that Ogre tends to overestimate the bonding distance at the interface due to the inability of its simplified electrostatic potential to fully capture interactions that are quantomechanical in their essence fully. For visualization purposes, in Figure 2 of the Main text we corrected such distance manually by taking as a reference the Pb-I bond length found in the two bulk materials (~3.2 Å). The raw output structures proposed by Ogre are attached as a part of the supplementary material.

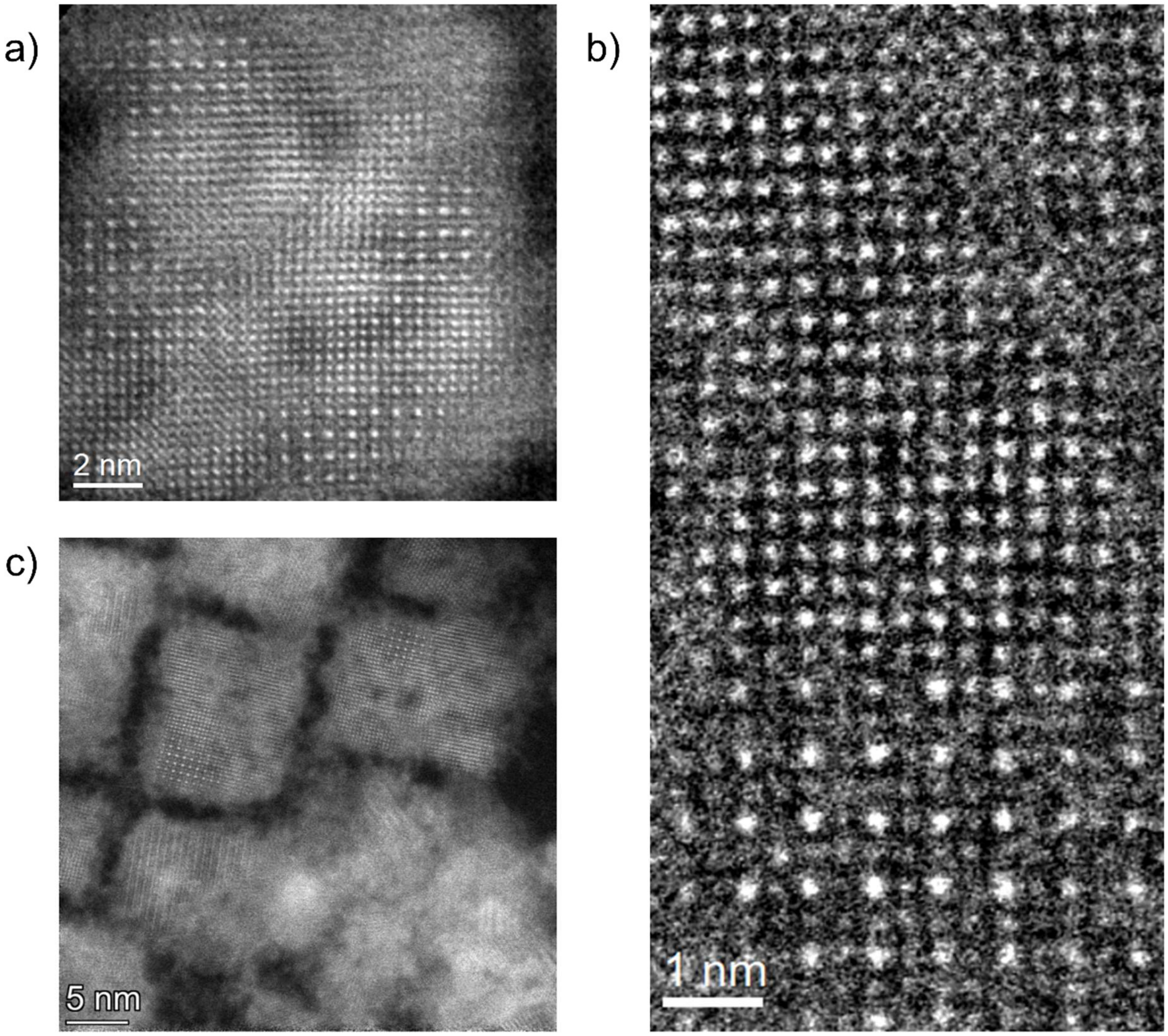

Figure S12. HAADF images of (a) $(EA_xCs_{1-x})PbI_3$ perovskite domains together with trigonal PbI_2 domain in the single NC. The formation of $(EA_xCs_{1-x})PbI_3/PbI_2$ heterostructures can be explained by the instability of the $EAPbI_3$ phase under an electron beam. (c) Lower magnification image of the $(EA_xCs_{1-x})PbI_3/PbI_2$ heterostructures. Figure 2b shows an atomic resolution HAADF image of the interface between $(EA_xCs_{1-x})PbI_3/PbI_2$ domains. The upper part of the images corresponds to the trigonal PbI_2 lattice at [-22-3] projection; it is connected to the $(EA_xCs_{1-x})PbI_3$ domain. Due to the very high beam sensitivity of the material, this analysis was made only qualitatively based on the intensity of the corresponding atomic columns. In the HAADF image the brightest columns

correspond to Pb/I due to the highest average Z number. The minimum contrast corresponds to higher EA containing (EA_xCs_{1-x}) columns. With an increase in Cs doping the contrast of (EA, Cs) columns is increased. The I columns in the ((EA_xCs_{1-x})PbI_3) have a contrast similar to the (EA, Cs) columns enriched in Cs, due to the little difference in Z numbers of I and Cs.

Table 1. Extracted lattice parameters from XRD patterns of the $EA_xFA_{(1-x)}PbI_3$ NCs. The corresponding composition (here indicating the fraction of EA^+ and FA^+ present in the alloy), was estimated by applying Vegard's law: $x = (d_x - d_B)/(d_A - d_B)$, where d_x, d_A, and d_B are the measured lattice constants of the alloy and of the pure materials *A* and *B*, respectively.

Material	Lattice constant (Å)	Composition ($EA_xFA_{(1-x)}PbI_3$)
$EAPbI_3$	6.4308	1.00
$EAPbI_3$ + 7 µL FA-oleate	6.3980	0.61
$EAPbI_3$ + 14 µL FA-oleate	6.3919	0.54
$FAPbI_3$	6.3460	0.00

Table 2. Extracted lattice parameters from XRD patterns of the $EA_xFA_{(1-x)}PbI_3$ NCs. The corresponding composition (here indicating the fraction of EA^+ and FA^+ present in the alloy) was estimated by applying Vegard's law: $x = (d_x - d_B)/(d_A - d_B)$, where d_x, d_A, and d_B are the measured lattice constants of the alloy and of the pure materials *A* and *B*, respectively.

Material	Lattice constant (Å)	Composition ($EA_xCs_{(1-x)}PbI_3$)
$EAPbI_3$	6.4308	1.00
$EAPbI_3$ + 3.5 μL Cs-oleate	6.3722	0.73
$EAPbI_3$ + 7 μL Cs-oleate	6.3481	0.62
$EAPbI_3$ + 14 μL Cs-oleate	6.2786	0.29
$CsPbI_3$	6.2151	0

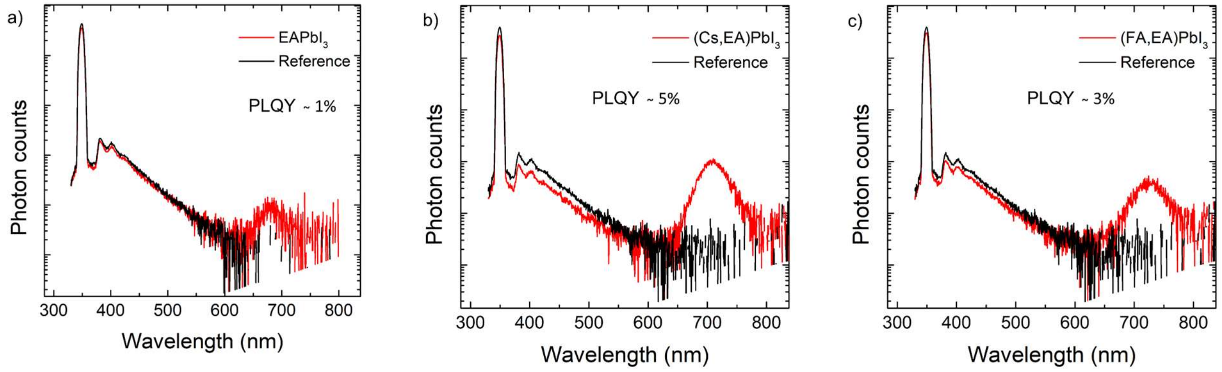

Figure S13. Spectra recorded for PLQY measurement of (a) $EAPbI_3$, (b) $(Cs, EA)PbI_3$, and (c) $(FA, EA)PbI_3$ perovskite NCs, respectively.

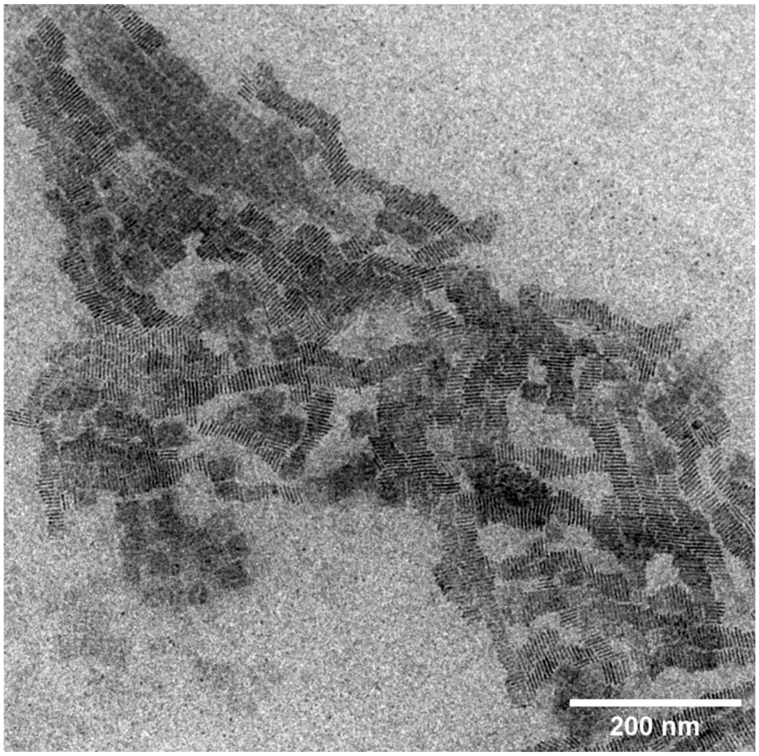

Figure S14. (a) Low magnification and TEM images of the 2 monolayers $EAPbI_3$ nanoplatelets.

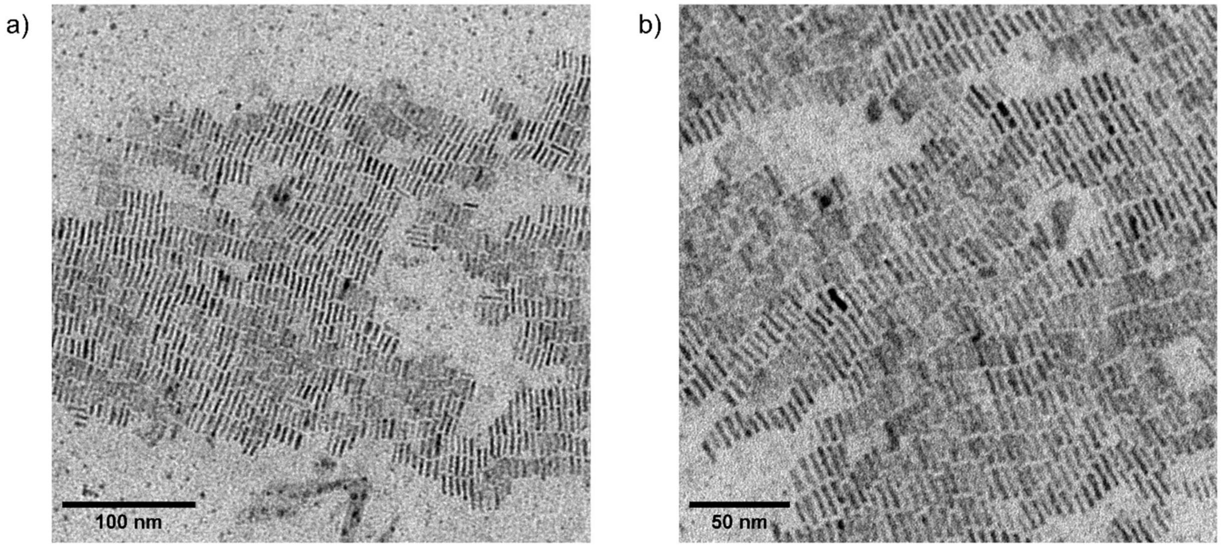

Figure S15. (a, b) Bright field TEM images of the mixed thickness $EAPbI_3$ nanoplatelets in different magnifications.

S3. $EAPbI_3$ nanoplatelets analysis

The XRD pattern of $EAPbI_3$ nanoplatelets shown in Figure 4d demonstrates the characteristic features of multilayer diffraction, that is a secondary interference effect occurring in XRD patterns of highly ordered assemblies of colloidal nanocrystals. The phenomenon is described in detail in Refs.[6,7] , but in short it consists in the formation of sharp fringes due to the constructive interference of radiation scattered by neighboring particles. For nanoplatelets, this XRD profile can be simulated, and in the best cases refined, using the code developed by us in Ref.[6].

Prior to the analysis, the pattern must be prepared by performing a manual background subtraction (Figure S16, top), followed by the subtraction of residual peaks that do not belong to the equally spaced series of multilayer interference fringes (Figure S16, bottom). In this specific case, two of the residual peaks ($q = 1.0$ Å^{-1} and $q = 2.0$ Å^{-1}) belong to $EAPbI_3$ nanoplatelets that are positioned perpendicular to the silicon substrate, and therefore do not take part to multilayer interference, while the peak at 2.2 Å^{-1} is a spurious reflection from the substrate. From the $EAPbI_3$ residual peaks we can extract the lateral lattice periodicity (and therefore Pb-Pb distance), that is 6.35 Å.

Following the background and spurious peak subtraction, the XRD pattern was analyzed using our code from Ref.[6] Although the quality of data was insufficient for a detailed refinement of the nanoplatelets structure, which would allow to precisely determine the position of all heavy atoms, the XRD profile is fully compatible with a thickness of 2 PbI_6 octahedra, corresponding to a nominal stoichiometry of $(OLAM)_2EAPb_2I_7$. The nanoplatelets stacking periodicity was estimated from the fit to 39.5 Å, while the disorder parameter is estimated to $\sigma_L = 0.8$ Å (see Ref.[6] for a full description of the method and parameters).

Another relevant parameter extracted from the fit is the vertical distance of the two Pb^{2+} ions layers found in the platelet structure. This was found to be 6.31 Å, which is similar to the horizontal Pb-Pb distance in the same platelets (see above) and much smaller compared to the Pb-Pb distance in $EAPbI_3$ cuboidal nanocrystals. We clarify that the intensity profile in Figure S16 appears different from both Figure S15 and Figure 4 of the Main Text (note the intensity ratio of peaks in the 0.5 – 1.25 $Å^{-1}$ and 1.5 – 2.25 $Å^{-1}$ range) because our data analysis script works by fitting the square module of the structure factor (shown here), and not the experimental intensity (shown in the main text). The two profiles can be interconverted via the Lorentz-Polarization correction factor.[8]

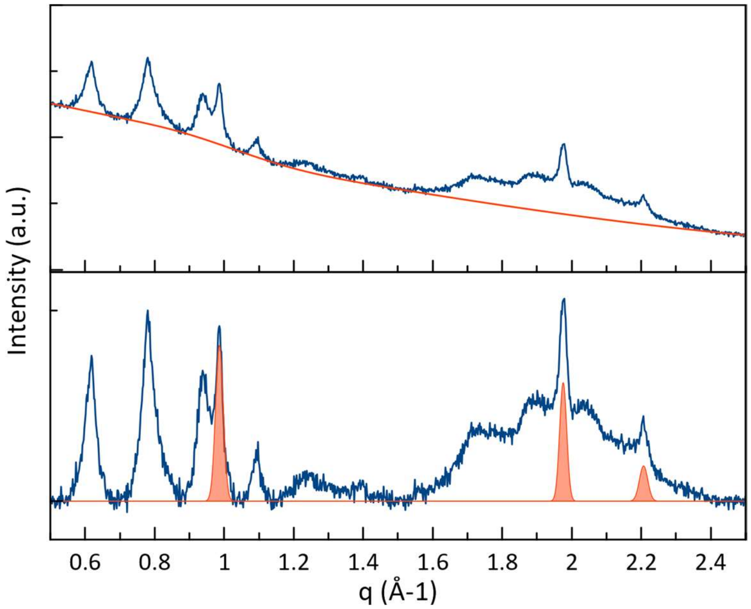

Figure S16. XRD pattern of EAPbI3 nanoplatelets plotted in *q*-scale ($q = 4\pi \sin(\theta)/\lambda_{X\text{-ray}}$), with background highlighted (top) , and after background subtractions with residual peaks highlighted (bottom). manual background subtraction (top). The pattern resulting from the subtraction of both background and residual peaks has been used as an input for the multilayer diffraction analysis script from Ref.[6] (see Figure S16).

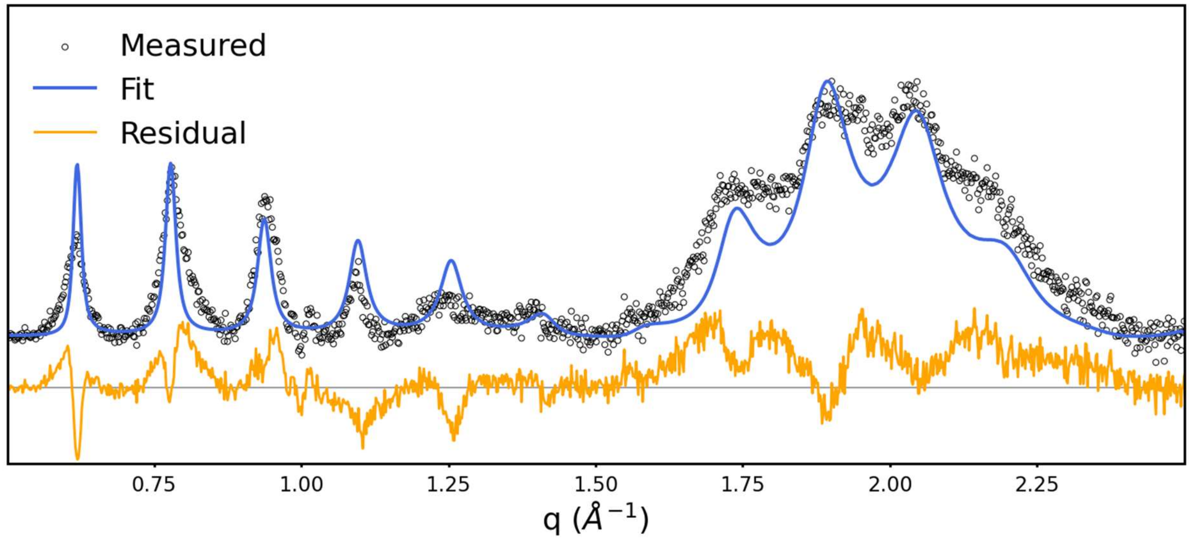

Figure S17. Multilayer diffraction simulation of the $EAPbI_3$ platelets XRD pattern. The high signal/background ratio does not allow a high quality refinement, but the simulation is sufficient to estimate the platelets stacking periodicity and vertical Pb-Pb distance with decent accuracy.

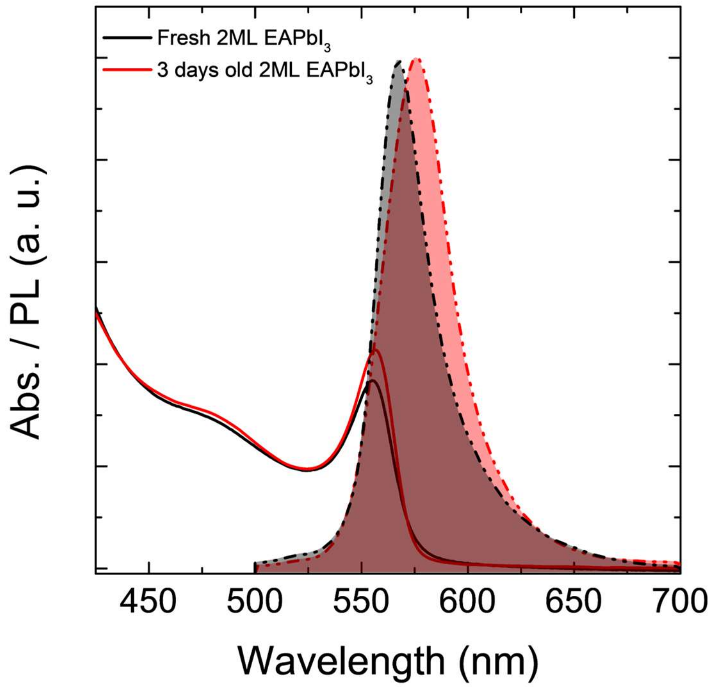

Figure S18. Optical absorbance and PL spectra of the 2 ML $EAPbI_3$ nanoplatelets as prepared (black) and after 3 days of aging (red) under ambient conditions in a closed vial.

S4. References

(1) Muralidhar, J. R.; Salikolimi, K.; Adachi, K.; Hashizume, D.; Kodama, K.; Hirose, T.; Ito, Y.; Kawamoto, M. Chemical Storage of Ammonia through Dynamic Structural Transformation of a Hybrid Perovskite Compound. *J Am Chem Soc* **2023**, *145* (31), 16973–16977. https://doi.org/10.1021/jacs.3c04181.

(2) Toso, S.; Dardzinski, D.; Manna, L.; Marom, N. Fast Prediction of Ionic Epitaxial Interfaces with Ogre Demonstrated for Colloidal Heterostructures of Lead Halide Perovskites. https://doi.org/10.26434/chemrxiv-2024-hwthh.

(3) Moayedpour, S.; Dardzinski, D.; Yang, S.; Hwang, A.; Marom, N. Structure Prediction of Epitaxial Inorganic Interfaces by Lattice and Surface Matching with Ogre. *Journal of Chemical Physics* **2021**, *155* (3). https://doi.org/10.1063/5.0051343.

(4) Moayedpour, S.; Bier, I.; Wen, W.; Dardzinski, D.; Isayev, O.; Marom, N. Structure Prediction of Epitaxial Organic Interfaces with Ogre, Demonstrated for Tetracyanoquinodimethane (TCNQ) on Tetrathiafulvalene (TTF). *The Journal of Physical Chemistry C* **2023**, *127* (21), 10398–10410. https://doi.org/10.1021/acs.jpcc.3c02384.

(5) Rothmann, M. U.; Lohmann, K. B.; Borchert, J.; Johnston, M. B.; McKenna, K. P.; Herz, L. M.; Nellist, P. D. Atomistic Understanding of the Coherent Interface Between Lead Iodide Perovskite and Lead Iodide. *Adv Mater Interfaces* **2023**, *10* (28). https://doi.org/10.1002/admi.202300249.

(6) Toso, S.; Baranov, D.; Giannini, C.; Manna, L. Structure and Surface Passivation of Ultrathin Cesium Lead Halide Nanoplatelets Revealed by Multilayer Diffraction. *ACS Nano* **2021**, *15* (12), 20341–20352. https://doi.org/10.1021/acsnano.1c08636.

(7) Toso, S.; Baranov, D.; Filippi, U.; Giannini, C.; Manna, L. Collective Diffraction Effects in Perovskite Nanocrystal Superlattices. *Acc Chem Res* **2023**, *56* (1), 66–76. https://doi.org/10.1021/acs.accounts.2c00613.

(8) Fullerton, E. E.; Schuller, I. K.; Vanderstraeten, H.; Bruynseraede, Y. *Structural Refinement of Superlattices from X-Ray Diffraction*; 1991; Vol. 45.
